# Incorporating wrestling into physical education curriculum for the development of physical activity and anti-bullying intervention

**DOI:** 10.3389/fpsyg.2025.1643481

**Published:** 2025-08-12

**Authors:** Wan Peng, Longfei Wang, Quanzhi Li

**Affiliations:** Sports Training Academy, Nanjing Institute of Physical Education and Sports, Nanjing, China

**Keywords:** PA, wrestling, physical education curriculum, physical activity, anti-bullying intervention

## Introduction

Physical activity (PA) has positive effects on adolescents in terms of physical, psychological, and social development. It helps enhance cardiovascular function, alleviate psychological stress, and improve social adaptability. According to the World Health Organization (WHO) standards, only 21% of boys and 16% of girls worldwide meet the daily recommended 60 min of moderate-to-vigorous physical activity per day. Moreover, PA levels decrease non-linearly during adolescence, with an annual decline rate of 7% between the ages of 13 and 15. The rate of decline in girls is 18% higher than that of boys (Marques et al., 2020), this trend is associated with the inefficiency of school physical education programs. As one of the primary avenues for adolescent physical activity engagement, physical education (PE) classes provide systematic training. Although different sports in PE classes have varying impacts on adolescents' physical and mental health, the primary goal remains the enhancement of overall wellbeing. However, a single course intervention can only increase PA levels by 17%, while a comprehensive intervention combining community resources can achieve up to 47% improvement (Kriemler et al., 2011; Casey et al., 2014).

Cross-national study indicate that traditional bullying manifestations (e.g., physical aggression) have decreased in most countries, while cyberbullying has significantly increased since 2018 (Molcho et al., 2025). A survey of 1,454 American adolescents aged 12–17 revealed that 72% of respondents had experienced cyberbullying at least once in the past year, with 85% also having experienced school bullying (Juvonen and Gross, 2008). An Irish study with a sample of 2,474 adolescents found that ~10.83% considered themselves victims of cyberbullying, and 5.15% saw themselves as perpetrators (Corcoran et al., 2015). In high-income countries, the rate of cyberbullying is as high as 16.2%, with 9% experiencing both online and offline bullying, particularly among adolescents with obesity (who show 30% higher risk of dual-victimization; Nguyen et al., 2024). School bullying causes irreversible harm to adolescents' physical and mental health, leading to endocrine disorders, somatic symptoms, depression, anxiety, and suicidal tendencies. The lifetime treatment cost for depression and anxiety disorders in bullying victims is $12,500 per person, 83% higher than that of the general population (Moore et al., 2017). Annually, 19.7% of global new cases are attributed to bullying-related risks (Biswas et al., 2020). Students with stronger athletic abilities are often more popular and less likely to become targets of bullying. Long-term physical activity has been shown to inhibit aggression by improving empathy (understanding of other people's emotions), reducing shyness and increasing self-esteem (Peiyan, 2019), students who are excluded from physical education (such as those with specific learning disabilities) are more likely to be victims or perpetrators of bullying, and systematic exclusion “increases the risk of bullying and creates conditions for bad social behavior” (Jesina et al., 2022), Insufficient physical activity in PE classes may lead to a range of mental health issues, increasing the risk of bullying, Physical inactivity is positively correlated with anxiety and depression, and psychological vulnerability amplifies the negative effects of life events (such as bullying; Wang et al., 2025).

Wrestling is a combative sport that generally strengthens adolescents' physical fitness and psychological development. Research shows that with wrestling training, adolescent athletes experienced a 5.33% decrease in body fat percentage (Enling et al., 2023), a 9% increase in basal metabolic rate (Xingfei et al., 2006), and adolescents aged 15–16 undergoing wrestling training demonstrated a 10.55% increase in strength, a 4.58% increase in agility, a 3.89% increase in speed, and a 9.34% improvement in overall physical fitness (Звонарьов and Сватьєв, 2024). A 10-week traditional Chinese wrestling training program with 40 college students aged 18–22 showed significant reductions in perceived stress and depressive symptoms (Amini et al., 2014). Moreover, wrestling is beneficial in terms of physical contact, rule awareness, and self-protection. The safety fall training in wrestling shows that the body contact training can effectively enhance the sports adaptability (Gasienica-Walczak et al., 2010), Yi wrestling passes on culture and strengthens discipline through regulated competitions during traditional festivals (such as Torch Festival and Spring Festival; Yan et al., 2019), gamified simulations of falling scenes are just as effective as rigorous training in improving self-protection skills (Gasienica-Walczak et al., 2010). Wrestling helps reduce the likelihood of becoming a victim or perpetrator of bullying, research has shown that mandatory judo courses in Japanese middle schools including wrestling techniques have reduced violent crime rates among 15–18-year-old males by 41% (Melendez-Torres et al., 2023). In a pilot program at a high school in Shenzhen, China, wrestling helped release stress with cortisol levels decreasing by 19% and simulated conflict scenarios using virtual reality systems, reducing students' anxiety scores by 1.2 standard deviations. Additionally, a “cross-grade mixed teams” model older students guiding younger students in training increased the social network density index from 0.31 to 0.48, reducing isolated nodes by 34% (Lucas Miranda da Silva et al., 2021). In summary, wrestling education uses simulated combat scenarios to release dopamine, cultivates empathy through role rotation between attacker and defender, enhances self-protection abilities, and helps prevent school bullying behaviors.

## Fundamental considerations for integrating wrestling into physical education classes

According to the definition by the United World Wrestling (UWW), wrestling is a competitive activity in which the athlete controls and throws the opponent to the ground through technical actions during close combat. The technical system of wrestling includes throws, joint locks, pins, escapes, and other techniques, from its origins as an official event in the ancient Olympic Games to its present status as a core event in the modern Olympics, wrestling has always maintained the purity of competitive sports, showcasing the richness of various national sports cultures while also reflecting the differences among various ethnic cultures. Therefore, integrating wrestling into physical education classes can promote physical fitness, technical skills, intelligence, and even ideological development, it also serves as an important platform and pathway for cultural recognition and inheritance (Liang, 2022).

Wrestling is an ideal comprehensive physical exercise suitable for introduction into physical education classes. It combines the cultivation of skills, strategies, and psychological qualities, which traditional physical training may not provide. First, traditional physical training typically includes strength training, weightlifting, endurance training on top of these basic exercises, improves agility, coordination, an reaction speed through confrontational exercises, which are apart of its unique qualities. Evidence demonstrates that wrestling significantly improves performance metrics (e.g., 60-meter sprint, pull-ups and the 3 × 10–meter shuttle run), its effects are superior to traditional physical training, as confrontational actions such as grappling technique, activate full-body muscle coordination, particularly emphasizing the role of core muscles in dynamic stability (Zhengyu, 2018). Second, students learn to form rule-transfer abilities through the study of wrestling rules, which can be transformed into moral qualities in daily life. The two-person confrontation in wrestling creates appropriate pressure situations. Through technical confrontations of “control and counter-control” such as the positional struggle in Chinese–style wrestling, students can gradually develop risk management abilities and emotional regulation skills (Meng et al., 2024). Additionally, wrestling training enhances both physical fitness and self-confidence in combat, leading to a positive self-defense ability (Dongfeng, 2020). Third, wrestling has a long history and fully reflects the rich diversity of ethnic cultures. Its history can be traced back to prehistoric civilizations and has evolved into unique cultural forms across different ethnic groups around the world. Tibetan wrestling in China reflects the respect for the land among highland ethnic groups (Hongfang, 2020), Uyghur wrestling reflects an awareness of risk avoidance, Japanese sumo has a strict training system, and the ranking of sumo wrestlers mirrors the class hierarchy in feudal society (Yuan et al., 2024). Mongolian wrestling costumes feature ribbons that symbolize the honor of warriors, and Uzbek wrestling belts are embroidered with tribal totems. From the ceremonial characteristics of Chinese-style wrestling to the spirit of the Mongolian wrestling of the steppes and the Central Asian wisdom of Uzbek wrestling, this sport has become a living carrier of ethnic cultural genes.

**Figure 1:**
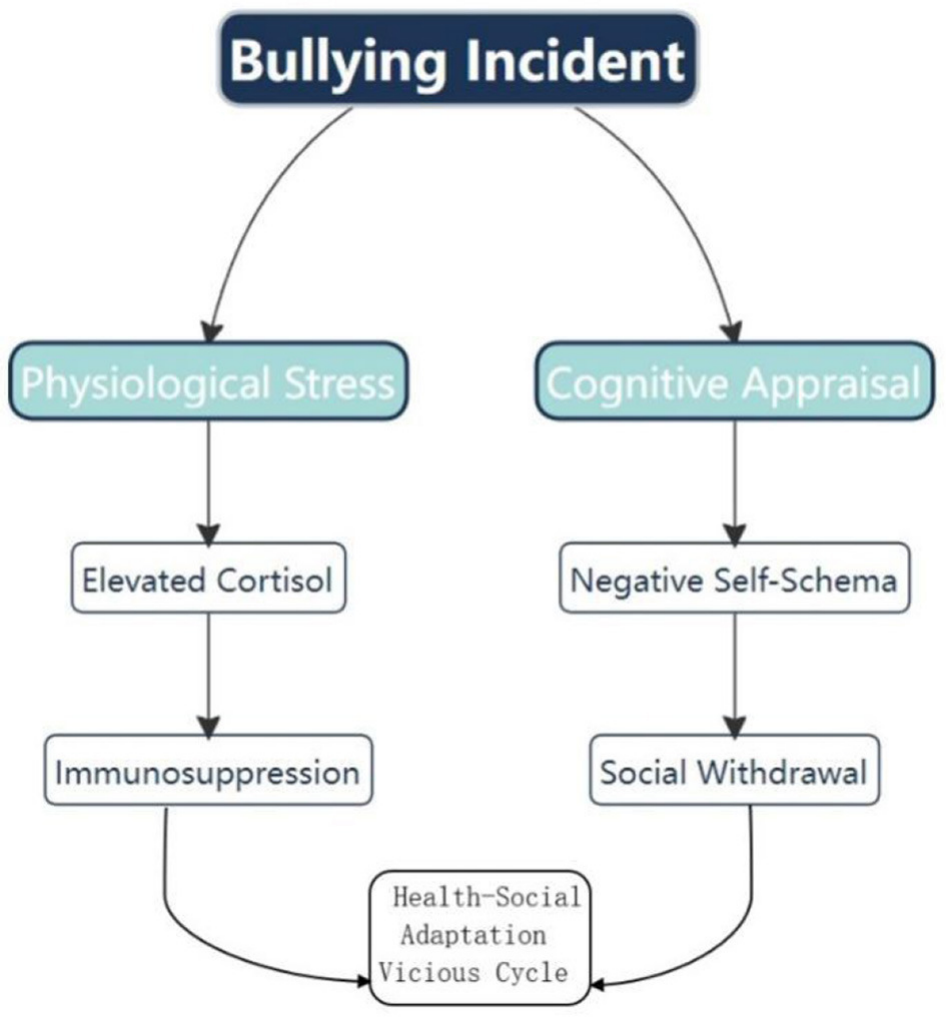
” The relationship between bullying incidents and health-social adaptation.

In summary, the three-dimensional synergistic mechanism of wrestling, from “combative physical development to psychological resilience cultivation, and then to cultural identity construction,” demonstrates the unique value of this sport (Figure 1).

## Empirical foundations for integrating wrestling into PE

The physical education curriculum establishes a tripartite synergistic mechanism utilizing wrestling training as the medium. This mechanism is founded upon: (1) the enhancement of physical fitness, (2) the cultivation of psychosocial competencies, and (3) the preventive effect on school bullying. Its core mechanism lies in the dual enhancement of physical competence and psychosocial adaptation. This manifests as a dual-path framework: one path builds deterrence through improved physical capabilities, while the other suppresses aggression through enhanced psychosocial competencies. Concurrently, it incorporates dual-risk-reduction mechanisms—both direct and indirect—for mitigating bullying: specifically, reducing students' bullying perpetration and decreasing their victimization risk through wrestling training. Crucially, the curriculum design emphasizes leveraging the inherent advantages of wrestling while ultimately serving the primary objective of “preventing school bullying.”

First, the direct impact of wrestling on students' physical fitness. Research on wrestling's impact on strength quality shows that an 8-week functional strength training program for male freestyle wrestlers increased peak torque of flexor and extensor muscles by 10–15%, indicating improved muscle balance (Jianli, 2022). A 12-week core training program for female wrestlers resulted in significant improvements, with bench press increasing by 14.2%, clean and jerk by 12.5%, and enhanced core stability contributing to better technical performance (Fengming, 2020). Another study on 15–17-year-old high school boys showed that wrestling training significantly improved anaerobic power which is related to short–term high–intensity exercise ability and increased circumference measurements of shoulder and chest muscles (Genç, 2020). Research on explosive power and speed demonstrated that freestyle wrestling experimental group students performed significantly better than the control group in tests such as the 60-meter sprint, pull-ups, and 3 × 10-meter shuttle runs, indicating that wrestling directly promotes explosive power and movement speed (Marques et al., 2020). A Ukrainian study found that students involved in wrestling training performed better in the 100-meter sprint and standing long jump, proving that it enhances lower limb explosive power (Griban et al., 2021). Research on flexibility and coordination showed that in Chinese-style wrestling teaching, strength training for the waist, abdomen, and legs was proven to enhance body control, while confrontational exercises significantly improved reaction speed and coordination (Ziyan, 2022). An experiment at Grozny High School showed significant progress in seated forward bend tests for wrestling group students, indicating targeted development of flexibility (Zhengyu, 2018). In terms of body composition, a study on 42 male participants showed that after wrestling training, their BMI significantly decreased while muscle mass increased, indicating the beneficial regulatory effect of wrestling on body composition (Pangemanan, 2020).

Second, the potential impact of wrestling on psychological and social adaptation. (1) Development of Willpower and Confidence. Research indicates that resilience training programs that cultivate psychological qualities can reduce bullying (Wang et al., 2023). Therefore, wrestling courses can integrate resilience training, such as setting challenging goals, encouraging perseverance, and enhancing students' psychological resilience. Wrestling training, through repeated encounters with failure and pressure, helps students build a resilient mindset. For example, Chinese students reported that “facing a powerful opponent requires calmness and an unwillingness to give up.” This psychological resilience may reduce the risk of becoming a bullying victim. Studies on Brazilian Jiu-Jitsu show that wrestlers perform better in emotional control and responsibility awareness such as self–managing weight, which is closely linked to improved social adaptation (Thomas and Zamanpour, 2018). (2) Discipline and Team Identity. Wrestling courses, when integrated with ideological education, can cultivate students' willpower, characterized by “bravery, tenacity, and perseverance,” which represents a form of discipline recognition. A 16-week wrestling program with 70 primary school students resulted in a 37% increase in team belonging scores (MacPhail et al., 2004). In a study involving 200 participants in a “throwing” project, 41% of participants believed that wrestling's most significant impact was on “developing team spirit,” followed by enhancing interpersonal trust (Jianhong, 2012).

Finally, wrestling training directly and indirectly helps prevent campus bullying. Direct Association Studies primarily focus on wrestling's dual impact on aggression and bullying. (1) Evidence of Suppressed Aggressive Behavior. Chinese martial arts training including wrestling techniques through philosophical education and skill practice significantly reduced both reactive aggression (passive counterattacks) and proactive aggression (deliberate harm), the intervention group showed a 32% reduction in aggressive behavior. In combined Brazilian Jiu-Jitsu and wrestling training, students reduced aggressive behavior by 41% through rule internalization such as respect in opponent sand prohibiting malicious strikes and emotional management exercises (Ting and Guodong, 2019). High-frequency ≥4 times per week physical exercise, especially competitive sports, may increase aggression in boys. If the wrestling course design emphasizes competition over cooperation, this could exacerbate this risk. However, if it integrates teamwork and emotional regulation training, it may help suppress aggression (Méndez et al., 2019). (2) Potential Risk Disputes. A comparative study by Mutz and others found that among male middle school students participating in wrestling and boxing, the acceptance of aggressive behavior was 27% higher than that of participants in non-contact sports, and physical aggression increased by 19%. This could be linked to the competitive training model or the coach's lack of emphasis on moral constraints (Mutz, 2012).

Indirect Association Studies focus on wrestling's multi-dimensional pathways to reduce bullying. Building Physical Fitness to Lower the Risk of Becoming a Victim. Research shows that boys with lower physical fitness such as poor sprinting speed are more likely to become victims of bullying, while physical fitness advantages can reduce the risk. Wrestling, as an exercise to enhance fitness and agility, theoretically helps disadvantaged boys reduce their chances of being targeted. However, excessive competition may lead to the opposite effect. The influence of fitness on girls' victimization risk is not significant, and intervention should focus more on social relationships and emotional management, with less direct correlation to wrestling (Han et al., 2025). Therefore, wrestling courses may offer protective effects for disadvantaged boys, but teaching methods for girls may need adjustment, focusing on cooperation rather than confrontation. A study of college students showed that for every 1-point increase in physical self-efficacy, the likelihood of excellent physical health increased by 10.9%. Wrestling training, through mastering techniques, boosts confidence and reduces victimization risk by promoting protective behaviors (Ziqing et al., 2023), enhancing Psychological Endurance to Resist Bullying. Research shows that students participating in wrestling had an 18.6-point increase in their Rosenberg Self-Esteem Scale scores (maximum score 40), significantly higher than the control group *p* < 0.01. Through simulated failure training in confrontational scenarios such as countering after being pinned, students improved their emotional regulation, and anxiety levels when during bullying exposure decreased by 34% (Xiufeng, 2013). Improving Peer Relationships and Group Identity, studies show that physical exercise, through improving emotional management and easing interpersonal relationship issues, indirectly reduces bullying behavior. If wrestling is combined with emotional regulation training, coach–guided conflict reflection, this approach may enhance students' social competence and mitigate bullying perpetration (Zhang and Deng, 2024).

## Discussion

Wrestling, as a unique sport with high metabolic demands and structured social interaction, offers an innovative solution to the public health dilemma of insufficient adolescent physical activity PA and the coexistence of campus bullying. This study demonstrates that integrating wrestling into physical education curricula, coupled with appropriate teacher guidance, interventions are made in three aspects: physical, psychological and social adaptation ability of students, this approach not only reduces victimization incidence by increasing students' time spent in moderate-to-vigorous physical activity (MVPA), but also mitigates aggressive behavior through the enhancement of emotion regulation skills and social competencies. However, wrestling classes inherently carry a higher risk of injury. Students may sustain short-term injuries due to technical errors during practice. Consequently, both parents and school administrators may express resistance to such courses. If instruction focuses solely on technique while neglecting mental discipline, the potential for aggressive behavior could increase. Therefore, the value of wrestling within physical education curricula is not absolute but contingent upon whether the pedagogy effectively channels primal aggressive impulses into self-mastery, so course designs should emphasize non-competition and education. The core value of this paper lies in transforming traditional “aggressive” confrontational behaviors into embodied educational tools—developing functional strength through mechanical control exercises to eliminate “victim physique” and cultivating responsible autonomy under rule constraints to disrupt the bullying cycle.
